# On Investigating the Microstructural, Mechanical, and Tribological Properties of Hybrid FeGr1/SiC/Gr Metal Matrix Composites

**DOI:** 10.3390/ma14010174

**Published:** 2021-01-01

**Authors:** Eugene E. Feldshtein, Larisa N. Dyachkova, Justyna Patalas-Maliszewska

**Affiliations:** 1 Institute of Mechanical Engineering, University of Zielona Góra, 65-516 Zielona Góra, Poland; E.Feldshtein@iim.uz.zgora.pl; 2 Powder Metallurgy Institute, Belarusian National Academy of Sciences, 220005 Minsk, Belarus; Dyachkova@tut.by

**Keywords:** hybrid metal matrix composites, graphite, silicon carbide, microstructure, mechanical properties, tribological behavior

## Abstract

In recent years, studies of different properties of hybrid metal matrix composites, as well as very detailed issues, have been published. In this article, ready-made iron, graphite, and silicon carbide powders were used to produce the base material and composites. An analysis of some microstructural and mechanical properties, as well as the tribological behavior of metal matrix composites (MMCs), based on FeGr1 sintered material with the single and hybrid addition of a silicon carbide and graphite was undertaken. During the study, the flexural and compressive strength of MMCs were analyzed and changes of the momentary coefficient of friction, the temperature of friction, as well as wear rates of the MMCs tested were monitored. Based on the results, it was revealed that wear rates decreased 12-fold in comparison to the base material when SiC or SiC + Gr were added. Further research into MMCs with ceramic particle additives is proposed.

## 1. Introduction

Metal matrix composites are various classes of materials that consist of a metal matrix, usually reinforced with a ceramic component in the form of particles, whiskers, and short and long fibers, which have been successfully used for many years in various branches of industry. Iron, copper, aluminum, magnesium, and titanium alloys and many other metals are used as the base metal matrix, and carbides, oxides, borides of various chemical elements, solid lubricants, and other substances are used as reinforcing compounds. Silicon carbide (SiC) holds an important place among the substances mentioned above.

Xia et al. [1] investigated the fatigue properties of Al-2124A composites with the addition of 25% SiCp of 3 microns, in size and registered the improvement of fatigue characteristics in comparison with unreinforced material. Detailed fractography revealed the presence of unique, cone-shaped cracks in samples that break down under high cycle fatigue. Rofman et al. [2] analyzed the properties of semi-solid, AA2024 alloy containing fine-grained β-SiCp particles or mechanically alloyed granules. Sub-micron particles did not significantly increase the hardness of composites. At enhanced temperatures, tensile testing showed increases in the ductility and crack resistance of AA2024 + SiCp compared to the initial material. Ponhan et al. [3] studied Mg-based nanocomposites reinforced with 10, 20 and 30 wt.% SiC nanoparticles. As the content of SiC nanoparticles and the grinding time increased, finer and more equiaxial nanocomposite powders were obtained. The relative density and hardness of the Mg-SiC sintered samples were improved when the grinding time was increased. Kumar et al. [4] analyzed Mg-based composites. It was noted that the introduction of solid ceramic particles such as Al_2_O_3_, SiC, B_4_C, TiO_2_, and TiC increased strength and hardness but reduced plasticity. Titanium increased the strength and the ductility of metal matrix composites (MMCs) tested. Huang et al. [5] studied the effect of the plastic deformation process on the evolution of the microstructural distribution and mechanical properties of magnesium-based composites reinforced with SiC particles. The uniform distribution of matrix grain sizes and the segregation of SiC particles along matrix grain boundaries were shown. Prasad et al. [6] examined the influence of the TiB_2_ and SiC adding on the microstructure and the mechanical and tribological properties of Al6061 MMC. The uniform distribution of TiB_2_ and SiC particles and the intermetallic Mg_2_Si phases in an aluminium matrix were established. Microhardness increased after the addition of TiB_2_ particles to the matrix. This addition also improved the wear resistance of composite samples, which was due to their self-lubricating properties. Lee et al. [7] investigated aluminum composites with a high volume fraction of SiC particles. Larger reinforcement provided increased wear resistance, as this helps MMCs maintain rigidity under severe, shear deformation. In addition, a modification of the SiC interface increased the wear resistance of composites by preventing the formation of interfacial cracks between the matrix and reinforcing particles. Tosun et al. [8] tested Al-Mg MMC reinforced with microdimensional SiC and Al_2_O_3_ particles at different volume ratios. The highest porosity coefficient for all test conditions was 17%; the lowest porosity coefficient was 5.4%. A generally homogeneous microstructure was observed. Saranu et al. [9] noted that composites based on Mg alloy have lower density, higher strength properties, and higher wear resistance, and they can be reinforced with different ceramic particles. It has been noted that hybrid composites are characterized by low weight, high strength, hardness, wear resistance, and fatigue. Patel et al. [10] developed Al matrix composite materials reinforced by 5% SiCp particles. It was shown that under all loading conditions, MMCs improved tribological properties compared to un-reinforced AA5052; the addition of SiCp increased the density of AA5052. Surya et al. [11] described the microstructure and mechanical characteristics of MMCs based on Al7075 reinforced with SiCp. SEM analysis registered the uniform scattering of SiC particles with smaller number of pores. A composite with 15 wt.% SiC has higher mechanical properties, hardness, strength, and bending strength in comparison with other MMCs. Kumar et al. [12] compared samples of Al5052 and Al7075 alloys in the initial state and after reinforcing with SiC and graphite. The results showed that Al-SiC-Gr is an exceptional combination of composites with a high strength-to-weight ratio. Mohanraj et al. [13] have produced composites based on AA6082 using SiC, Ti, Ni, and Cr. The results showed that the hardness and tensile strength increased in comparison with the base metal. Raheja et al. [14] described the effectiveness of MMC based on Al5086 alloy with additions of graphene and silicon carbide. Zhang et al. [15] studied the microstructural and mechanical characteristics of 6061 composite, based on aluminum reinforced with 10 vol.% SiC particles in comparison with 6061 monolithic alloy. The significant difference in microstructures of the materials compared was confirmed. Li et al. [16] created a model of the fatigue limit stress of different composites reinforced with carbon fibers and silicon carbide. 

Our research focused on analyzing the microstructural, mechanical, and tribological properties of FeGr1-based sintered MMCs with the single and hybrid addition of a silicon carbide and graphite. The results presented by Olejnik et al. [17] considered the technology of manufacturing TiC-FeCr local composite reinforcement in a cast steel matrix and the features of their macro- and microstructure, mechanical properties, and wear resistance. 

Two main area of analysis of the properties of metal matrix composites were studied in this work: (1) adding 1% graphite into the base material and (2) adding 1% silicon carbide.

Based on the analysis carried out, the need to examine the properties of metal matrix composites, based on FeGr1, was pointed out. Research materials and methods are described in Section 2, while details of the results of the research are provided in Section 3. Finally, the discussion has been provided, and the direction of further investigation is presented.

## 2. Materials and Methods

**Figure 1:**
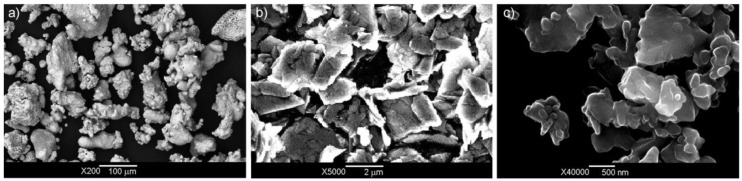
Iron (**a**), graphite (**b**), and silicon carbide powders (**c**).

Ready-made iron, graphite, and silicon carbide powders were used to produce the base material and composites. The iron powder particles had average dimensions of less than 200 µm, the graphite particles used both for the base material and for the composites had an average size of 3 µm, while the silicon carbide particles had an average size of ≈350 nm (Figure 1).

The single particles of silicon carbide formed conglomerates; in order to grind them into smaller particles, prior to the addition of the mixtures, ultrasonic action in alcohol was applied; they should be ground and then mixed with an iron powder and dried.

For MMCs production, particles of 1% Gr, 1% SiC, and hybrid (0.5% Gr + 0.5% SiC) were added to the base FeGr1 matrix. It was found in [18] that an increase in the additive content above 1% provided a deterioration of the composite properties, and the optimal percentage was 0.5%. Therefore, this percentage was adopted in this study.

The base material FeGr1 has been produced using iron powder and 1% graphite. The powders were placed in a “drunken barrel” device in which they were mixed for 3 h. Then, the powders were pressed using a hydraulic press with a pressure of 500 MPa. After in situ pressing, the samples were sintered in an electric furnace under 1100 °C for 1 h, and an endothermic gas atmosphere was used when sintering. The hardness of samples was HB 90 ± 2 for FeGr1 base material, HB 70 ± 2 for MMC with 1% Gr addition, HB 100 ± 3 for MMC with 1% SiC addition, and HB 98 ± 2 for MMC with hybrid addition, as it was described in [19].

Structures were studied with the metallographic microscope “MEF-3” (Reihert, Vienna, Austria). The fracturing features were tested using a “Mira LMH” scanning electron microscope (Tescan, Brno, Czech Republic). An ‘Oxford Instruments’ INCA 350 analyzer (Oxford Instruments Analytical Ltd, Bucks, England) was used for the SEM analysis. The X-ray diffraction method was applied to determine MMCs phase compositions and a “Rigaku” (Tokyo, Japan) ULTIMA IV X-ray diffractometer was used to accomplish this. Compounds were found in accordance with the “PDXL2” software package. The MMCs’ compressive and bending strengths were studied with the help of the “Tinius Olsen H150K-U” universal testing machine (Tinius Olsen TMC, Horsham, PA, USA). Compression tests were realized basing the [20] standard. Bending tests were realized basing the standard [21]. 

Tribological behavior was carried out using the own-made A-135 tester (Zielona Góra, Poland) based on the widespread roll–block scheme. Concentrated contact conditions were used when testing. Blocks (samples) were produced from the tested composites. The rolls (counter-bodies) were made of 41Cr4 steel with a hardness of 45–50 HRC. The tests were carried out using LAN-68 machine oil with an oil flow of 30 drops/min. A constant load of 1000 N was applied for a test period of 1 h. The linear speed in the contact zone was 0.45 m/s.

The operating surfaces of the samples were ground to ensure a surface roughness parameter Ra = 0.5 ± 0.02 μm.

The momentary coefficient of friction μ:

(1) $$\mu =\frac{2M}{FD_{o}}$$

where *M*—the moment of friction that was registered (Nm), *F*—the load (N), and *D*_o_—the roller diameter (mm).

The temperature in the friction zone was measured with a chromel–alumel thermocouple.

The volume of wear *I_V_*:

(2) $$I_{v}=\frac{D_{ol_{p}}^{2}}{8}\left\{2arcsin\frac{b}{D_{o}}-sin\left[2arcsin\frac{b}{D_{o}}\right]\right\},\;({\mathrm{mm}}^{3})$$

where $l_{p}$—the width of block (sample) (mm); *b*—the average width of the friction track (mm).

The wear rate $I_{V0}$:

(3) $$I_{Vo}=\frac{I_{V}}{L},\;({\mathrm{mm}}^{3}/\mathrm{km})$$

where *L*—the path of friction (km).

During the study, changes in the moment of friction and temperature in the friction zone were monitored, and the momentary coefficient of friction was then calculated. On the basis of the measurements of the width of the wear tracks, the value of volumetric wear and the wear rates were calculated. The Dini-Lite digital microscope with an accuracy of 0.001 μm was used to measure widths of tracks.

In order to ensure statistical significance, all measurements were carried out three times at each experimental point.

## 3. Results and Discussion

### 3.1. The Microstructure and New Compounds Characterization

**Figure 2:**
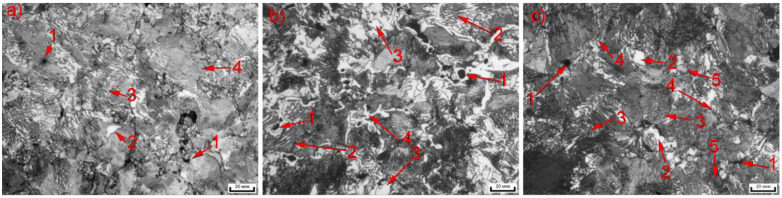
Microstructures of the metal matrix composites (MMCs) studied: (**a**) FeGr1, (**b**) FeGr2, (**c**) FeGr1 + 1% SiC, (1—pores, 2—ferrite, 3—lamellar pearlite, 4—granular pearlite, 5—SiC inclusions.

**Figure 3:**
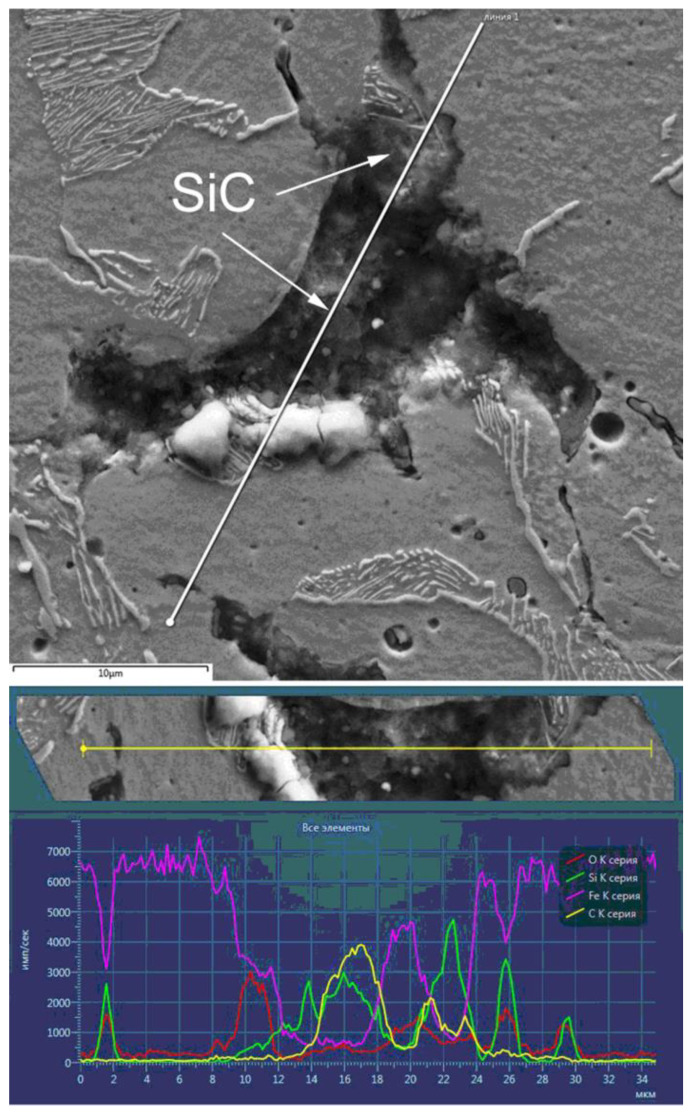
The distribution of elements in the MMC after the addition of 1% SiC.

A pearlite–ferrite structure characterizes the FeGr1 material (Figure 2a), and lamellar and granular forms are characterized for pearlite. The adding of 1% graphite provides pearlite forming in which the cementite torn mesh is observed; however, granular pearlite is absent. (Figure 2b). When adding 1% SiC, structural fragmentation appears, residual porosity increases, and the contents of the granular pearlite and the ferrite also increase considerably. The sizes of grains decreased 2–3 times. The finely dispersed inclusions in the structure can be also observed (Figure 2c). SiC particles are placed along the boundaries of the grains (Figure 3). This suggestion is substantiated by the structural fragmentation in MMCs, to which SiC’s have been added. In this case, ultrafine SiC particles prevent the growth of grains under secondary recrystallization.

The introduction of silicon carbide and graphite particles into the FeGr1 material resulted in forming new chemical compounds in the composites studied, namely α-SiC and SiC, as well as iron silicate Fe_2_(SO_4_) were registered. 

### 3.2. Strength of the Materials

**Figure 4:**
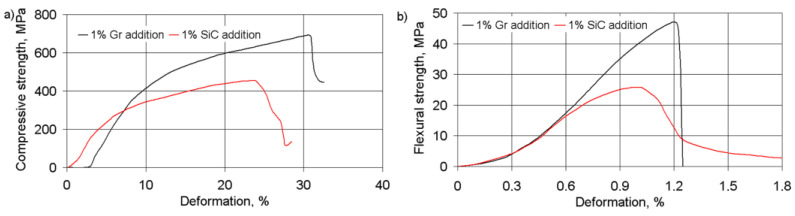
The stress–strain dependences for compression (**a**) and flexure (**b**) tests of materials with the addition of 1% Gr and 1% SiC.

The effects of particles added onto stress–strain dependences and flexural and compression stresses of the composite materials were tested. The stress–strain dependences for the materials studied are shown in Figure 4.

Compared to the addition of 1% graphite, the addition of 1% silicon carbides in a FeGr1 metal matrix decreases the ultimate stresses in the composite, while the degree of limiting deformation changes insignificantly. 

**Figure 5:**
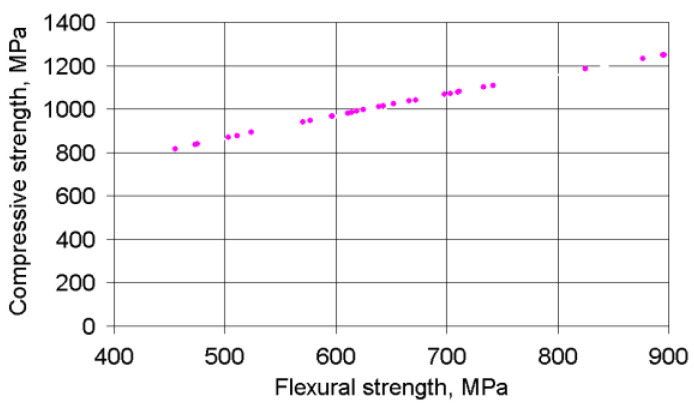
Correlation between compressive stresses and flexural stresses.

The interconnection between the ultimate compression and flexural stresses (Figure 5) can be described using the regression equation:

(4) $$\sigma_{c}=17.474\sigma_{f}^{0.728}\;(\mathrm{MPa})$$

The determination coefficient for this equation is equal to ≈1.0.

### 3.3. Features of Fracturing for the Materials Tested

The breaking of sintered materials occurs mainly along the grain boundaries, so the content and distribution of alloying elements, grain sizes, and the length of their boundaries play an essential role in the breaking features.

**Figure 6:**
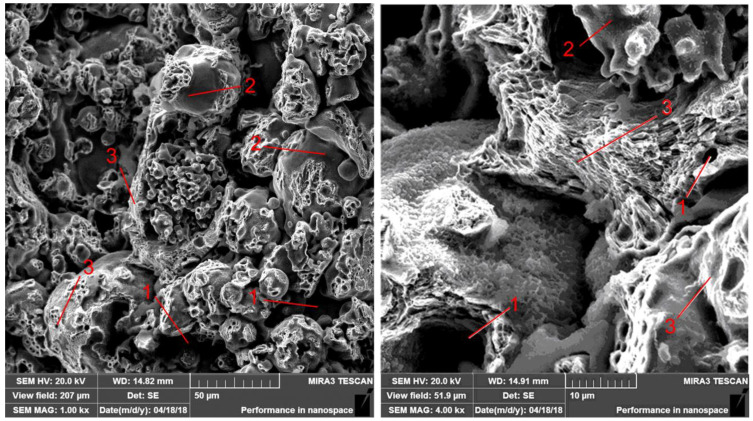
The fractures of FeGr1 base material.

When studying fractograms, it was found that fracturing in the base material is a low-power process because of the presence of numerous pores (1) at the particle joints, in which the initial porosity reached 15–17%. At the location of pores, there are no contacts between the particles, so there are relatively smooth surfaces (2) of their fracture. In areas where ferrite is predominant, viscous fracturing (3) is observed, while in areas where pearlite is predominant, quasi-brittle fracturing is observed (Figure 6).

**Figure 7:**
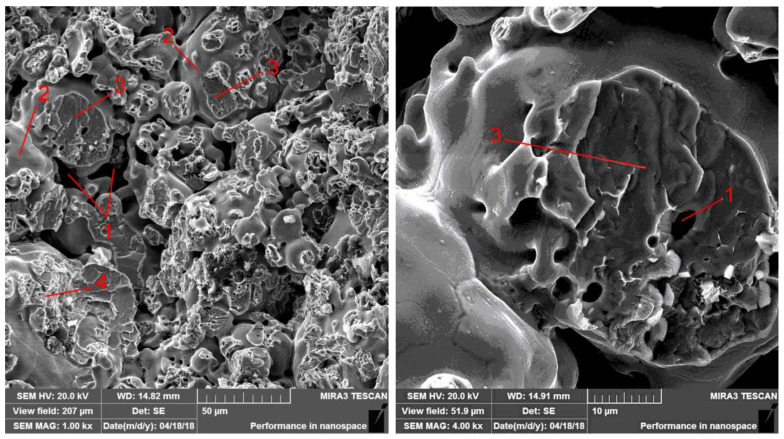
Fracturing of iron-based material with the addition of 1% graphite.

When adding 1% graphite into the base material, there is some increase in the amount of metal contact; this is due to the acceleration of diffusion processes on account of the reduction in the temperature of phase transformations in areas with high carbon content. In this case, the structure of the material consists of a stronger phase of pearlite and a harder phase of cementite; i.e., the strength of the material is higher than that of the base material. The fracturing mechanism is characterized by higher energy consumption and the occurrence of brittle fracturing. Inclusions of pores (1), smoothed areas of inter-granular fracturing (2), facet trans-crystalline fracturing (3) in places of cementite location, and quasi-brittle fracturing (4) in pearlite areas are registered in the fractograms (Figure 7).

**Figure 8:**
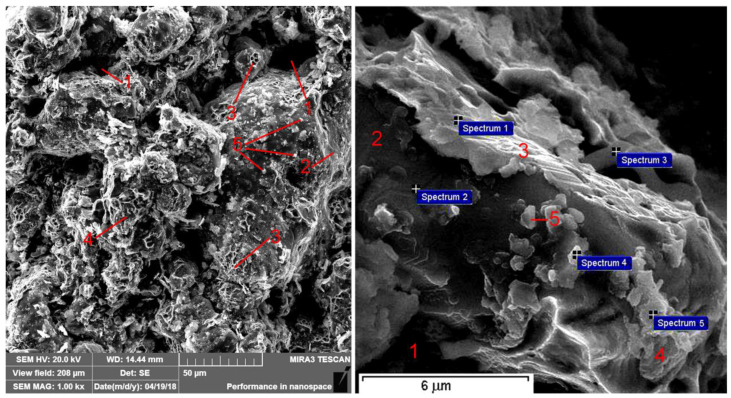
Fracturing of iron-based material with the addition of 1% of silicon carbides.

The addition of silicon carbide (Figure 8), where the particles are located on the grain boundaries, leads to a decrease in the strength of the same; this reduces the strength of the material, reduces energy consumption, and changes the character of the destruction. Pores (1) are observed upon fracturing, as is the case of the base material and material with the addition of graphite. At the same time, the size and number of smooth areas of inter-granular fracturing (2) reduce due to the presence of a large number of finely dispersed silicon carbide particles (5). Since the amount of pearlite in the structure decreases and the amount of ferrite increases, there is an increase of dimpled, viscous fracturing (3) in areas where ferrite is present and a decrease in the amount of quasi-brittle fracturing (4) in areas where pearlite is present. The distribution of elements at the surface of the fracture is presented in Table 1.

### 3.4. Tribological Behavior of the Materials Tested

**Figure 9:**
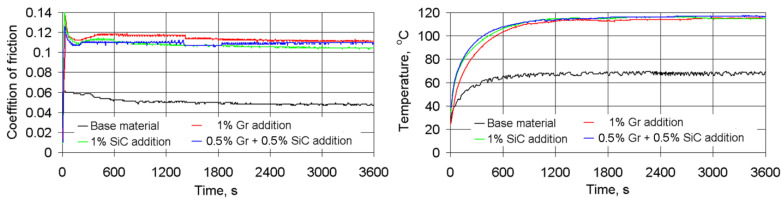
Changes in the time of the momentary friction coefficient and the friction temperature.

**Figure 10:**
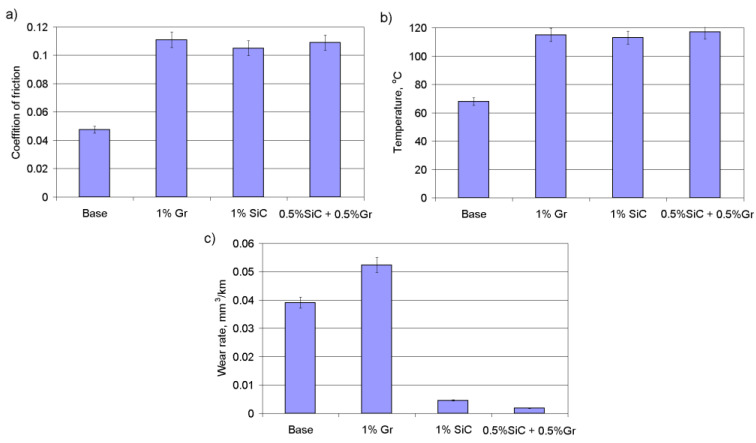
The effects of additive composition on the momentary coefficient of friction (**a**), friction temperature (**b**) and wear rate (**c**).

A comparison of the momentary coefficients of friction and temperatures in the friction zone revealed that for the composites tested, the coefficients of friction and temperature were approximately twice as high when compared with the base material (Figure 9). The running-up time is relatively short and does not exceed 15–20 min. The composition of used additives scarcely changes the momentary coefficient of friction but does cause changes in temperature levels by some 15–20%, with the difference in the wear rate reaching 12 times (Figure 10). Such a significant increase in wear resistance is due to the specific nature of the wear of the composites studied.

It is easy to observe that in the case of the base material (a), typical signs of adhesive wear and, to a lesser degree, abrasive wear can be observed. With the additional introduction of graphite, the content of pearlite in the composite, i.e., the content of iron carbide particles, increases, which intensifies abrasive wear, while the intensity of the adhesive processes decreases (b); highly characteristic features of the wear of composites in the case of the addition of SiC (c) or in the case of the hybrid addition of SiC + Gr (d) are observed.

SEM analysis (Table 2, Table 3, Table 4 and Table 5) revealed the presence of silicon carbides on the friction surfaces and also oxides, which was caused by high temperatures in the friction zone; the presence of dissolved silicon in the iron matrix was also registered. In the process of friction, oil molecules enter the pores of the material and contribute to the self-lubricating effect, as is evidenced by the presence of sulfur.

**Figure 11:**
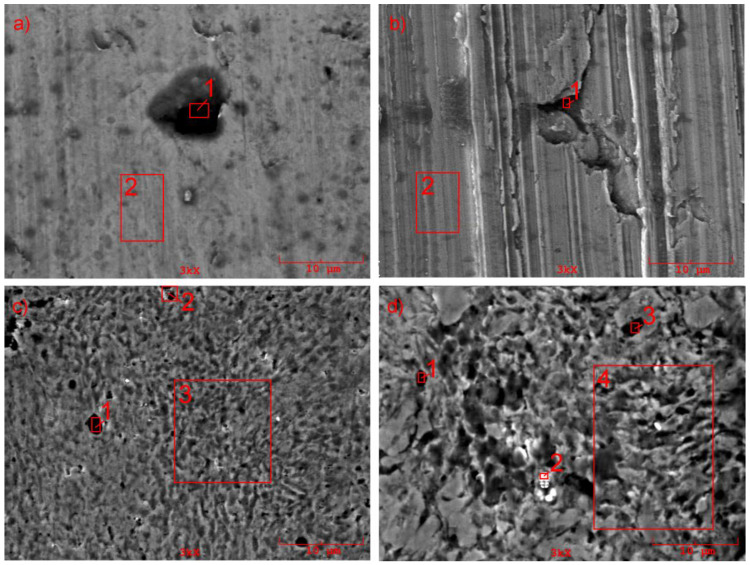
Worn surfaces of materials tested: (**a**) the base material, (**b**) the base material with the addition of 1% Gr, (**c**) the base material with the addition of 1% SiC, (**d**) the base material with the hybrid addition of Gr and SiC; 1- Spectrum 1 in Table 5; 2-Spectrum 2 in Table 5; 3- Spectrum 3 in Table 5; 4 - Spectrum 4 in Table 5.

As described in [22], the wear behavior of composites under significant loads is characterized by the specific nature of the wear. Under such conditions and regardless of the composition of MMCs or of the methods of reinforcement and manufacturing techniques on the friction surfaces of composites, a complicated spongy-capillary texture is formed (see Figure 11c,d), providing a significant increase in the wear resistance. The tribolayer formed contains oil molecules, iron matrix fragments, and solid strengthening particles, providing the effective workability of the friction pair.

## 4. Conclusions

In this investigation, an analysis was undertaken of some of the microstructural and mechanical properties as well as the tribological behavior of MMCs based on FeGr1 sintered material, with the addition of hybrid silicon carbide and graphite. Based on the results, the following were found:

- An addition of 1% graphite to the base sintered material forms a pearlite structure with a cementite torn mesh when compared to the initial, pearlite–ferrite structure. When 1% SiC is added, structural fragmentation occurs, and the content of granular pearlite and ferrite increases significantly; silicone carbides are also located along the boundaries of the grain.
- Compared to the addition of 1% graphite, the addition of 1% silicon carbides to the FeGr1 metal matrix leads to a reduction in the ultimate stresses in the material.
- Depending on the composition of the additive, various conditions occur in the fracturing of the material; particularly prevalent are viscous fracturing, inter-granular fracturing, facet transcrystalline fracturing, dimpled viscous fracturing, and/or quasi-brittle fracturing.
- With regard to the composites tested, the coefficients of friction and temperature were approximately twice as high when compared to the base material. The composition of the additives hardly changed the momentary coefficient of friction, changing the temperature level by some 15–20% only, but wear rates decreased 12 times as much when compared to the base material once SiC or SiC + Gr had been added; this is due to the formation of a spongy-capillary texture on the friction’s surface. This effect is followed by changes in the microstructure, the local hardness, and the fatigue strength of the MMCs, as well as the admission of the liquid lubricant into the micropores.

In summing up, it is to be emphasized that the addition of a hybrid of graphite and silicon carbide particles improves all the most important characteristics of composite materials; this is important for many industries and requires further analysis. Other ceramic particles such as nitrides, carbides, and oxides can be added to material and the properties analyzed could be extended to include plasticity, ductility, fatigue resistance, and so on.

## Figures and Tables

**Table 1:** The SEM point analysis of the FeGr1 + 1% SiC fractural submicroarea.

Spectrum Number	C, %	Si, %	Fe, %
Spectrum 1	21.5	2.2	The rest
Spectrum 2	47.9	0.3	The rest
Spectrum 3	26.0	2.7	The rest
Spectrum 4	61.3	11.6	The rest

**Table 2:** Compositions of elements in microareas of the base material.

Spectrum Number	Elements, %			
	C	O	S	Fe
Spectrum 1	36.77	3.6	0.7	58.93
Spectrum 2	1.63	7.35	0.45	90.57

**Table 3:** Compositions of elements in microareas of material with the addition of 1% Gr.

Spectrum Number	Elements, %			
	C	O	S	Fe
Spectrum 1	18.47	7.78	0.48	73.27
Spectrum 2	15.79	6.71	0.38	77.12

**Table 4:** Compositions of elements in microareas of material with the addition of 1% SiC.

Spectrum Number	Elements, %				
	C	O	S	Fe	Si
Spectrum 1	13.83	7.29	0.31	54.46	23.3
Spectrum 2	2.87	24	0.34	64	8.79
Spectrum 3	1.37	11.1	0.63	85.37	1.53

**Table 5:** Compositions of elements in microareas of material with the hybrid addition of Gr and SiC.

Spectrum Number	Elements, %				
	C	O	S	Fe	Si
Spectrum 1	6.1	17.86	2.55	65.11	8.38
Spectrum 2	10.32	2.69	0.46	29.29	57.24
Spectrum 3	7.11	28.86	2.88	59.45	1.7
Spectrum 4	1.6	11.84	5.43	78.61	2.52

## Data Availability

The data presented in this study are available on request from the corresponding author.
